# A Dual Adaptive Interaction Click-Through Rate Prediction Based on Attention Logarithmic Interaction Network

**DOI:** 10.3390/e24121831

**Published:** 2022-12-15

**Authors:** Shiqi Li, Zhendong Cui, Yongquan Pei

**Affiliations:** School of Computer and Control Engineering, Yantai University, Yantai 264005, China

**Keywords:** feature interaction, recommendation system, attention mechanism, logarithmic networks, Newton’s identity

## Abstract

Click-through rate (CTR) prediction is crucial for computing advertisement and recommender systems. The key challenge of CTR prediction is to accurately capture user interests and deliver suitable advertisements to the right people. However, there are an immense number of features in CTR prediction datasets, which hardly fit when only using an individual feature. To solve this problem, feature interaction that combines several features via an operation is introduced to enhance prediction performance. Many factorizations machine-based models and deep learning methods have been proposed to capture feature interaction for CTR prediction. They follow an enumeration-filter pattern that could not determine the appropriate order of feature interaction and useful feature interaction. The attention logarithmic network (ALN) is presented in this paper, which uses logarithmic neural networks (LNN) to model feature interactions, and the squeeze excitation (SE) mechanism to adaptively model the importance of higher-order feature interactions. At first, the embedding vector of the input was absolutized and a very small positive number was added to the zeros of the embedding vector, which made the LNN input positive. Then, the adaptive-order feature interactions were learned by logarithmic transformation and exponential transformation in the LNN. Finally, SE was applied to model the importance of high-order feature interactions adaptively for enhancing CTR performance. Based on this, the attention logarithmic interaction network (ALIN) was proposed for the effectiveness and accuracy of CTR, which integrated Newton’s identity into ALN. ALIN supplements the loss of information, which is caused by the operation becoming positive and by adding a small positive value to the embedding vector. Experiments are conducted on two datasets, and the results prove that ALIN is efficient and effective.

## 1. Introduction

The performance of a recommendation system goes hand in hand with the interests of advertisers, publishers, and users. The cost-per-click (CPC) advertisement charging pattern [1], based on the number of clicks, has become popular for online advertisements. In other words, the more clicks, the higher the publisher’s revenue, and the better promotion effect and the greater potential revenue that the advertiser can obtain. Moreover, good recommendation performance can lead to the recommendation of suitable items for users in special contexts [2], which further enhances user satisfaction. Among many recommender systems, such those for online advertisements [3], news displays [4,5,6], and shopping recommendations [7,8,9], the click-through rate (CTR) plays an important role. The goal of CTR is to predict the probability that users click specific items through information about user profiles, item attributions, and contextual scenarios. There are multi-field categorical features in CTR prediction, representing a difference from computer vision and natural language processes, which have many continuous features. There are a large number of features in CTR datasets. Therefore, the performance of CTR prediction is limited in only applying individual features. Modeling complex feature interactions plays a key role in the success of CTR prediction [10]. Depending on their domain knowledge, experts manually select feature combinations at an early stage, which entails extensive costs in terms of manual labor and finance. In order to solve the above-mentioned problem, a factorization machine (FM) [11] was proposed to capture second-order feature interaction in the manner of the vector inner product. A field-aware factorization machine (FFM) [12] proposed field-aware embedding for fine-grained feature interaction, meaning that one feature should model multi-feature presentation for different fields. Based on FM, the attention factorization machine (AFM) [13] added an attention score calculated by an attention mechanism to every second-order feature interaction. A higher-order factorization machine (HOFM) [14] proposed high-order feature interactions based on FM and was a high-order version of FM. Mutual information between fields and labels was considered in the field-weighted factorization machine (FwFM) [2], which weighted every field compared to FM. All the models mentioned above learn low-order feature interactions. Nevertheless, high-order feature interactions should be considered to improve the accuracy of CTR prediction.

With the success of deep learning in computer vision [15,16,17] and natural language processes [18,19], many deep neural network (DNN)-based methods, such as the convolutional neural network, self-attention network, recurrent neural network, and graph neural network, were applied for feature interaction in CTR prediction. Wide & Deep [20] combine logistic regression (LR) [21] and deep neural networks (DNNs) for CTR prediction, which not only retain memorability but also attain generalizability via the wide component and the deep component, respectively. Furthermore, DeepFM [22] replaces the wide component with the FM component to learn both explicit and implicit feature interactions. Yang et al. [23] proposed an end-to-end transfer learning framework with fine-tuned parameters for CTR prediction. Lian et al. [24] proposed the compressed interaction network (CIN) to capture specific-order feature interactions. Concurrently, CIN was integrated with DNNs to capture both explicit and implicit feature interactions, which was called the extreme deep factorization machine (xDeepFM). Zhang et al. [25] proposed a novel framework called the multi-scale and multi-channel neural network (MSMC) to learn the feature importance and feature semantics for enhancing CTR prediction. Jose et al. [26] propose an interpretable, accurate, and efficient CTR estimator based on the neural additive factorization model (NAFM). However, these models conduct predefined order of feature interaction, which could introduce noisy interaction, degrading CTR prediction performance. Moreover, the relative importance of high-order feature interaction is not considered, which is important information for CTR prediction. For example, feature interaction {gender = female and genre = romance} would be more positive information than feature interaction {gender = female and language = Chinese} for predicting whether to watch a movie. To this end, the logarithmic neural network (LNN) [27] was introduced to capture adaptive-order feature interactions [28]. However, the input of LNN must be absolutized, which could cause a loss of information to some extent, and LNN adds a small value for zeros in embedding vectors, which introduces some noisy information. In order to solve these problems, we propose a novel model called the attention logarithmic network (ALN), which can adaptively model arbitrary-order feature interaction and learn the importance of high-order feature interaction. In addition, Newton’s identity [29,30] is integrated with ALN to reduce the loss of information caused by LNN. The ensemble model is called the attention logarithmic interaction network (ALIN).

The specific contributions are as follows:

1. We design a novel attention logarithmic network (ALN) to model adaptive-order feature interactions and distinguish the importance of different high-order feature interactions through the squeeze and excitation network (SENet);
2. The input of ALN must be positive, which could cause a loss of information. Thus, we integrate Newton’s identity modeling feature interactions with ALN to propose a new model called ALIN;
3. Comprehensive experiments on two datasets are conducted to show that our proposed model outperforms the state-of-the-art methods.

The rest of this article is organized as follows: in Section 2, we summarize the related work about CTR prediction and our proposed model. Section 3 provides a description of our proposed model in detail. In Section 3, we design elaborative experiments to present the superiority of our model and show the effects of hyper-parameters on two datasets. Several ablation experiments are performed to verify the effectiveness of the proposed component. Finally, the relevant conclusions are drawn in this paper.

## 2. Related Materials

As described above, great efforts have been made to improve the performance of CTR prediction by researchers and academics, both in the industry and in academia. CTR prediction has gradually developed from an FM-based shallow model to a DNN-based deep model. In this section, we briefly review past methods of feature interaction in CTR prediction. Knowledge relating to the proposed model, including information on LNN and Newton’s identity, is also briefly introduced.

### 2.1. Feature Interaction in CTR Prediction

Modeling feature interaction is an important task in CTR prediction, which has attracted huge attention both in academia and the industry. Logistic regression (LR) [21] is a linear approach that only models first-order feature interaction by way of weighted summation. FM learns the second-order feature interaction in the form of the vector inner product, which further improves the performance of modeling feature interaction. The field-aware factorization machine (FFM) considers field-award information and introduces field-aware embedding, which models multi-feature embedding for one feature. The attention factorization machine (AFM) takes the weight of second feature interaction into consideration, which learns the importance of second-order feature interaction through an attention mechanism. High-order factorization machines (HOFM) model high-order feature interaction, but they apply iterative computation to obtain high-order feature interactions, which consumes computational power and takes a lot of time.

Recently, many deep neural network-based approaches have been applied to CTR prediction. For example, Google proposed Wide & Deep [20], which combines LR and DNNs. Nevertheless, it retains the manual feature engineering in the LR component of this approach. Wide & Deep combines the advantages of memorability and generalization through the wide part and deep part, respectively. Deep & cross replaces the wide component in Wide & Deep with a novel component called CrossNet [31], which increases the degree of interaction between features. Similarly, DeepFM improves on the wide part of Wide & Deep by using the FM module to model explicit second-order feature interactions. PNN conducts product operation in the product layer to capture high-order feature interaction [32]. As in other models, the DNN layer is stacked on the product layer to learn implicit feature interactions. NFM is a neutralized version of FM, which replaces the second-order feature interaction with a DNN layer [33]. Nevertheless, these approaches enumerate all the feature interactions that could produce redundant information. Therefore, AFN was proposed by employing LNN to learn adaptive feature interactions. However, the input of LNN must be positive values, meaning that the embedding values may achieve absolute values and zero values should add a small value. This could disturb the information of raw embedding and increase noisy information for feature interaction. Additionally, the importance of feature interactions should be considered, since feature interactions play different roles in CTR prediction. In this work, we propose an attention logarithmic network (ALN), which considers the importance of high-order feature interactions and learns the adaptive order of feature interactions. Then, to compensate for the information loss caused by LNN, Newton’s identity is used as a complementary component modeling feature interaction called the attention logarithmic interaction network (ALIN).

### 2.2. Squeeze and Excitation Network

Hu proposed the squeeze and excitation network (SENet) [34], which adaptively recalibrates channel-wise feature responses by explicitly modeling interdependencies between channels. SENet consists of two phases: the squeeze phase and the excitation phase. The two phases are described in Section 3.3 in detail. SENet has many applications in the field of computer vision. Moreover, we apply SENet as a discriminator to distinguish the importance of feature interactions in this paper to achieve better CTR performance.

### 2.3. Logarithmic Neural Network (LNN)

**Figure 1:**
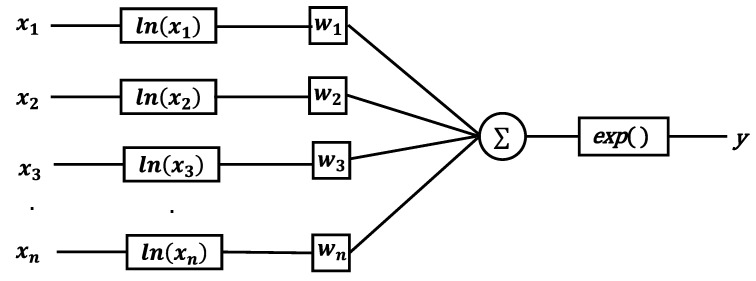
Logarithmic neuron.

There are many ways to approximate nonlinear functions [35]. Since muti-layer perceptron (MLP) cannot sufficiently approximate unbounded nonlinear functions, which requires a large number of parameters and has limited accuracy, LNN is proposed to fit unbounded nonlinear functions [27]. LNN consists of multiple logarithmic neurons. The structure of the logarithmic neurons is shown in Figure 1, where the original input is first transformed into logarithmic space, and then the output is obtained after weighted summation and exponential operation. Formally, the logarithmic neurons can be formulated as:

(1) $$y=exp\left(\overset{n}{\underset{i=1}{\sum }}w_{i}\ln x_{i}\right)=\overset{n}{\underset{i=1}{\prod }}x_{i}{}^{w_{i}}$$

Since MLP is not suitable for multiplicative, division, and exponential operations, it does not work well for fitting unbounded nonlinear functions. The operation of logarithmic transformation converts multiplication to addition, division to subtraction, and powers to multiplication by a constant. Therefore, LNN can fit unbounded nonlinear functions much better than MLP.

### 2.4. Newton’s Identity

Previous FM-based feature interaction models have modeled feature interactions in the form of symmetric polynomials whose complexity increases with the order of feature interactions. However, this complexity could reduce to linear time complexity by applying Newton’s identity [29,30] to feature interactions in the form of power sums.

## 3. Methods

**Figure 2:**
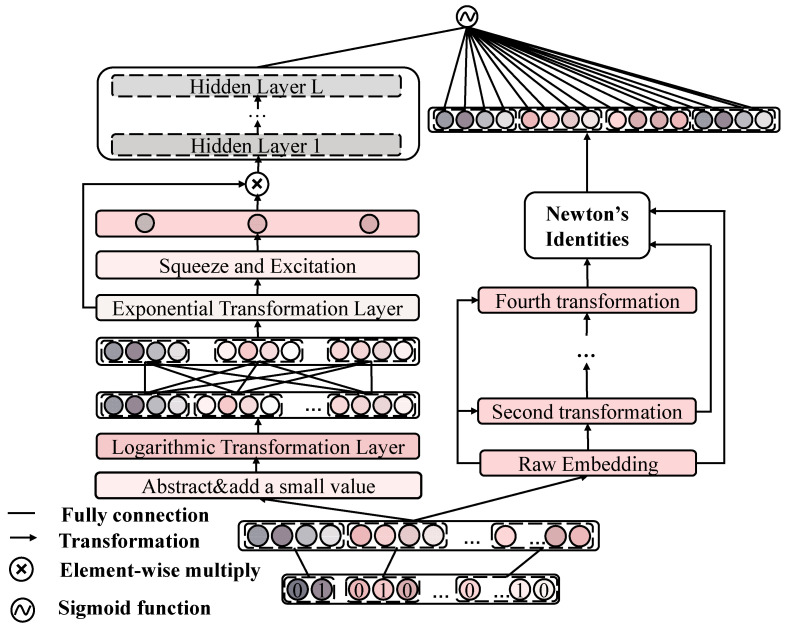
The architecture of proposed ALIN.

Before presenting the model in detail, we briefly summarize the proposed model named attention logarithmic interaction network (ALIN). First, the categorical features and the discretized continuous numerical features in the dataset are coded into one-hot vectors by a one-hot coding technique for easy input into the computer. Next, sparse one-hot features are converted to low-dimensional dense vectors by an embedding technique. Then, the embedding vector is passed into attention logarithmic network (ALN) and Newton’s identity component simultaneously. For further learning feature interactions, we stack multiple hidden layers on top of ALN. Finally, the out of hidden layer and Newton’s identity components are combined into sigmoid function to predict the click probability. The structure of ALIN is shown in Figure 2; the left half of the figure is the structure of ALN, and the right half is the Newton’s identity component. The specific implementation of each module is described in detail in the following sections.

### 3.1. Problem Definition

The CTR task is dedicated to predicting the click-through rate, which is the probability that a user will click on a specific item. Specifically, given a dataset, $D=\left\{\left(x_{1},y_{1}\right),\ldots ,\left(x_{s},y_{s}\right)\right\}$ containing s samples, where $x_{i}$ indicates the vector of user, item, and context information, and i indexes the samples. $y_{i}\in \left\{0,1\right\}$ is the ground truth of i-th sample. $y_{i}=1$ means a positive response from the user, such as clicking special advertisements or purchasing goods. Conversely, $y_{i}=0$ indicates that the user makes a negative response. The CTR task builds an efficient feature interaction model $f_{CTR}$ to take full advantage of user and item features x to predict the probability of click $\hat{y}$. The definition is given in Equation (2).

(2) $$f_{CTR}\;:x\;\to \;\hat{y}$$

### 3.2. Input Layer

Since the dataset for CTR prediction contains a large number of discrete features, one-hot coding of the raw features is required for input into the neural network. Suppose there are m different fields, each field may contain multiple features but each feature only belongs to one field. For example, one input instance $q_{i}$ could be coded as:

$$q_{i}=\left\{\underset{field\_1}{\underbrace{\left[0,1\right]}},\underset{field\_2}{\underbrace{\left[0,1,0\right]}},\underset{field\_3}{\underbrace{\left[0,0,0,1,0,0\right]}},\ldots ,\underset{field\_m}{\underbrace{\left[0,0,\ldots ,1,0\right]}}\right\}$$

there are *m* one-hot features and only one bit should be one in every one-hot feature.

### 3.3. Embedding Layer

**Figure 3:**
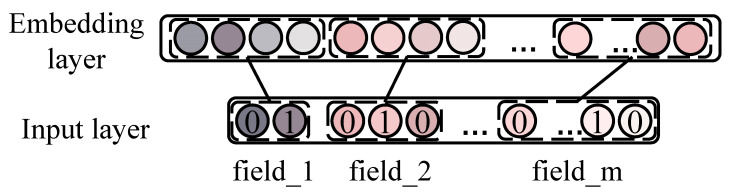
Structure of embedding layer.

The data in CTR prediction always contain multi-field categorical features, which are usually sparse and high dimensional after one-hot encoding. As in previous works, embedding technology is introduced to map these one-hot features into a low dimension and dense embedding vectors. As depicted in Figure 3, there are m feature fields who are independent of each other. First, the features in every field are transformed into high-dimensional and sparse feature vectors through one-hot encoding in the input layer. Then, the embedding layer is applied to one-hot encoding. Specifically, the embedding layer learns an embedding matrix for each field. Then, the embedding vector is queried by one-hot encoding. For example, the one-hot encoding of field ***i*** is $q_{i}$ and the corresponding embedding matrix is $V_{i}$. To obtain the embedding $e_{i}$, the following should be conducted:

(3) $$e_{i}=V_{i}q_{i}$$

where ***i*** indexes the fields. Similarly, the embedding vectors of all fields can be derived:

$$E=\left[e_{1},e_{2},e_{3},\ldots ,e_{m}\right]$$

where $e_{i}\in R^{d}$ represents the embedding of field *i*, and d indicates the dimension of embedding.

### 3.4. Attention Logarithmic Network

To learn the adaptive order of feature interactions and model the importance of high-order feature interactions adaptively, we propose an attention logarithmic network (ALN). As depicted on the left of Figure 2, the input of the logarithmic neuron must be positive. Therefore, the embedding vectors are applied to absolute value function and a small positive value (e.g., 1e-5) is added to zeros in embedding vectors. Consequently, the positive embedding can be represented as $\tilde{E}=\left[{\tilde{e}}_{1},{\tilde{e}}_{2},{\tilde{e}}_{3},\ldots ,{\tilde{e}}_{m}\right]$, which is used in successive layers.

**Figure 4:**
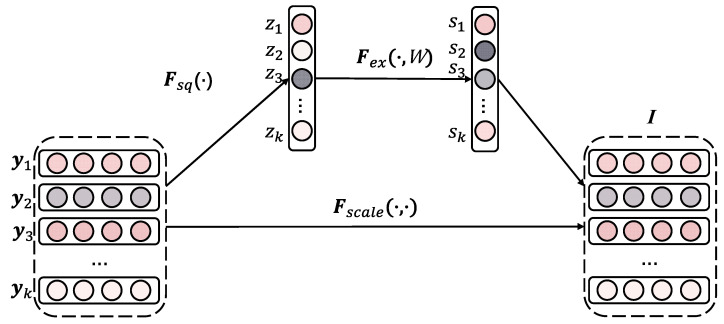
The squeeze–excitation mechanism used in our model.

Logarithmic neural network (LNN) and squeeze and excitation network (SENet) are important components of ALN, which learn the powers of logarithmic neurons also known as the orders of each feature in feature interaction and learn the importance of feature interactions, respectively. Unlike that in traditional LNN, the input of logarithmic neurons in ALN is vectors. To be more specific, the input of ALN comprises positive vectors $\tilde{E}$. Positive vectors $\tilde{E}$ are first transformed into logarithmic space by the logarithmic transformation layer. Then, weighted summation is conducted on the logarithmically transformed vectors in a vector-wise level. Finally, the result of weighted summation is converted into exponential space by the exponential transformation layer. The above operations can be formalized as follows:

(4) $$y_{j}=exp\left(\overset{m}{\underset{i=1}{\sum }}w_{ij}\ln\left({\tilde{e}}_{i}\right)\right)={\tilde{e}}_{1}^{w_{1j}}⊙{\tilde{e}}_{2}{}^{w_{2j}}⊙\cdots ⊙{\tilde{e}}_{m}{}^{w_{mj^{}}}$$

where *j* indexes the logarithmic neuron, $w_{ij}$ is the order of i-th field in *j*-th neuron, and ⊙ denotes element-wise product. According to Equation (4), ALN can learn feature interactions of an arbitrary order. For example, if $w_{1j}$ and $w_{2j}$ are equal to 1 and the other weighting coefficients are equal to 0, then the feature interaction $y_{j}={\tilde{e}}_{1}⊙{\tilde{e}}_{2}$ can be learned in j-th logarithmic neuron. However, the importance of feature interactions is not considered in Equation (4). Then, the SENet, which is stacked on the top of exponential transformation layer, is proposed to model the importance of feature interactions. The output of SENet layer can be formulated as follows:

$$I=\left[i_{1},i_{2},\ldots ,i_{k}\right]=\left[s_{1}y_{1},s_{2}y_{2},\ldots ,s_{k}y_{k}\right]$$

where $s_{i}\;$is the importance of the ***i***-th feature interaction, which is calculated from the squeeze–excitation mechanism, and *k* is the number of feature interactions as well as the number of logarithmic neurons. Next, we describe in detail how to calculate the feature importance factor $s_{i}$ via SENet. SENet contains two main stages including squeeze and excitation stages, which are described in Figure 4. In the squeeze step, all feature interactions are squeezed to summary information vector $Z=\left[z_{1},z_{2},\cdots ,z_{k}\right]$ by squeeze function $F_{sq}\left(\cdot \right)$, such as max pooling or mean pooling. If the mean pooling method is selected for calculating summary information, $z_{i}$ can be calculated as follows:

(5) $$z_{i}=F_{sq}\left(y_{i}\right)=\frac{1}{d}\overset{d}{\underset{t=1}{\sum }}y_{i,t}$$

where $i\in \left[1,\ldots ,k\right]$ and *d* are the dimension of embedding vector. In the excitation step, the summary information vector Z is passed into the two-layer perceptron, where the dimensionality is reduced in the first layer and the original dimensionality is restored in the second layer. Then, the importance scores are obtained. Formally, the importance scores can be formulated as:

(6) $$S=F_{ex}\left(Z\right)=\sigma_{2}\left(W_{2}\sigma_{1}\left(W_{1}Z\right)\right)$$

where $\sigma_{1}$ and $\sigma_{2}$ are activation functions and $W_{1}\in R^{k\times \frac{k}{r}}$, $W_{2}\in R^{\frac{k}{r}\times k}$ are weighted matrixes of two layers, and r is reduction ratio. Finally, the importance scores are multiplied by the original feature interaction to obtain the new feature interaction that distinguishes the level of importance. The new feature interactions can be calculated as follows:

(7) $$I=F_{\mathrm{scale}\;}\left(S,Y\right)=\left[s_{1}y_{1},s_{2}y_{2},\ldots ,s_{k}y_{k}\right]=\left[i_{1},i_{2},\ldots ,i_{k}\right]$$

where $s_{i}$ is a scalar value, $y_{i}\in R^{d}$ and $i_{i}\in R^{d}$.

To further enhance the feature interaction effect, multi-layer perceptron (MLP) is employed on top of importance-aware feature interactions. At first, all the importance-aware feature interactions are concatenated as the input of MLP:

(8) $$H_{0}=i_{1}⊕i_{2}⊕\ldots ⊕i_{k}$$

where ⊕ denotes concatenation operation. Then, $H_{0}$ is fed into MLP with ***H*** hidden layers:

(9) $$\begin{array}{c} H_{1}=Relu\left(W_{1}^{H}H_{0}+b_{1}\right)\\ \cdot \cdot \cdot \\ H_{l}=Relu\left(W_{l}^{H}H_{l-1}+b_{l}\right)\\ \cdot \cdot \cdot \\ H_{L}=Relu\left(W_{L}^{H}H_{L-1}+b_{L}\right) \end{array}$$

where $H_{l},W_{l}^{H}\;\mathrm{and}\;b_{l}$ are the output, weighted matrix, and bias vector of l-th hidden layer, respectively, and L is the number of hidden layers. The ALN module can stand independently as a CTR prediction model, but it still has some shortcomings that can be improved, which are described in detail below.

### 3.5. Newton’s Identity Component

Since the operations of taking absolute values and adding a small positive value to zeros of embedding vectors introduce noise to the embedded information, Newton’s identity is used to further model feature interactions in this paper. Analogous to FM, r-order feature interaction is modeled as follows:

(10) $$f_{r}=\underset{j_{1}>j_{2}>\cdots >j_{r}}{\sum }e_{j_{1}}⊙e_{j_{2}}⊙\ldots ⊙e_{j_{r}}$$

where $j_{i}$ is the ***i***-th index of r-order feature interaction. In this paper, Newton’s identity is applied to model high-order feature interactions in the form of power sums. Formally, the identity can be formulated as follows:

(11) $$f_{r}={\left(-1\right)}^{r}\underset{c_{1}+2c_{2}+\cdots kc_{k}=k}{\sum }\overset{k}{\underset{i=1}{\prod }}\frac{{\left(-p_{i}\right)}^{c_{i}}}{c_{i}!i^{c_{i}}}$$

(12) $$p_{i}=\overset{m}{\underset{l=1}{\sum }}{\left(e_{l}\right)}^{i}$$

where $p_{i}$ is the sum of the i-th power of the feature vector. The feature interactions from first order to fifth order based on Newton’s identity are concretely as follows:

(13) $$f_{1}=p_{1}$$

(14) $$f_{2}=\frac{1}{2}\left(p_{1}^{2}-p_{2}\right)$$

(15) $$f_{3}=\frac{1}{6}\left(p_{1}^{3}-3p_{1}⊙p_{2}+2p_{3}\right)\;$$

(16) $$f_{4}=\frac{1}{24}\left(p_{1}^{4}-6p_{1}^{2}⊙p_{2}+3p_{2}^{2}+8p_{1}⊙p_{3}-6p_{4}\right)$$

(17) $$f_{5}=\frac{1}{120}\left(p_{1}^{5}-10p_{1}^{3}⊙p_{2}+20p_{1}^{2}⊙p_{3}-30p_{1}⊙p_{4}-20p_{2}⊙p_{3}+15p_{1}⊙p_{2}^{2}+24p_{5}\right)\;$$

As in the above equation, Newton’s identity contains many terms of the sum of powers $p_{i}$. Thus, the intermediate variable $Q_{i}$ is introduced to facilitate the calculation of $p_{i}$. The intermediate variable $Q_{i}$ is defined as follows:

(18) $$\begin{array}{c} Q_{1}=E\\ Q_{2}=Q_{1}⊙E\\ \cdot \cdot \cdot \\ Q_{i}=Q_{i-1}⊙E\\ \cdot \cdot \cdot \\ Q_{R}=Q_{R-1}⊙E \end{array}$$

where ***R*** denotes the highest order of feature interaction. Through Equation (18), we can learn that the intermediate variable is initialized to the original embedding, i.e., $Q_{1}=E$; then, the new higher-order intermediate variable $Q_{i}$ is obtained by iteratively multiplying the current intermediate variable $Q_{i-1}$ with the original embedding vector E. Furthermore, the sum of power $p_{i}$ can be calculated by summing all rows in $Q_{i}$. Finally, the feature interactions from first order to R-th order are concatenated as:

(19) $$C_{R}=f_{1}⊕f_{2}⊕\ldots ⊕f_{R}$$

where ⊕ denotes concatenation operation.

### 3.6. Prediction Layer

In this layer, we first concatenate the output of ALN and Newton’s identity component:

(20) $$O=H_{L}⊕C_{R}$$

Then, the sigmoid function is employed to predict the click-through rate as follows:

(21) $$\hat{y}=\sigma \left(w_{o}O+b_{o}\right)$$

where $\hat{y}\in \left(0,1\right)$ is the predicted label of CTR, $w_{o}$ and $b_{o}$ are the weighted vector and bias vector of the prediction layer, respectively, and σ is the sigmoid function.

### 3.7. Optimization and Training

The CTR prediction problem is essentially a binary classification task. As in the work of previous researchers, the cross-entropy loss function is also adopted in our work. The cross-entropy loss function measures the distance between the ground truth and the predicted value as follows:

(22) $$J=-\frac{1}{N}\overset{N}{\underset{i=1}{\sum }}y_{i}\log\left({\hat{y}}_{i}\right)+\left(1-y_{i}\right)\log\left(1-{\hat{y}}_{i}\right)$$

where *N* is the number of training samples, $y_{i}$ is the true label of the *i*-th sample, and ${\hat{y}}_{i}$ is the prediction value of the *i*-th sample.

## 4. Results

In this section, we described extensive experiments that were conducted to verify the effectiveness of our model. Firstly, a brief overview of the dataset and experiment setup is presented. Then, comparative experiments are shown to demonstrate the effectiveness of the proposed model followed by hyper-parameter experiments to observe the effect of the hyper-parameters. At the end, several ablation experiments were conducted to verify the effectiveness of the individual components.

### 4.1. Datasets and Experiment Setup

#### 4.1.1. Datasets

1. **Criteo** (https://www.kaggle.com/datasets/mrkmakr/criteo-dataset, accessed publicly): Criteo is a famous benchmarking dataset for CTR prediction. It has 13 numerical feature fields and 26 categorical feature fields, all of which are anonymous feature fields;
2. **Criteo_600K**: We split 600 K samples from the full Criteo dataset randomly. It also includes 39 anonymous feature fields as in the full Criteo dataset.

In this paper, the numerical features are converted to categorical features that the numerical values z are transformed to $z=\lfloor ln^{2}\left(z\right)\rfloor$ if *z* > 2 and *z* = 1 otherwise. For example, when the numerical feature *z* = 10.5, the feature is first logarithmically transformed and then squared, and finally the floor operation is made, i.e., $z=\lfloor ln^{2}\left(10.5\right)\rfloor \;$= 5. Otherwise, when the numerical feature *z* = 1.5 (*z* ≤ 2), z is transformed to 1. Furthermore, all datasets are split into 8:1:1 for the train set, valid set, and test set, respectively. The details of the datasets are shown in Table 1.

#### 4.1.2. Evaluation Metrics

Following from previous work [10], we use three metrics, AUC (area under the ROC curve), log loss, and relative improvement (RI), to evaluate the proposed model.

1. ***AUC***: The *AUC* metric is widely used in CTR prediction, which measures the probability of a positive sample ranking higher than randomly chosen negative samples [36]. The larger the *AUC* value, the better the CTR effect. Moreover, the upper limit of *AUC* is 1. The definition of *AUC* is as follows:
  (23) $$AUC=\frac{\sum_{i=1,j=1}^{i\leq M,j\leq N}\delta \left(r_{i}-r_{j}>0\right)}{M\times N}+\frac{\sum_{i=1,j=1}^{i\leq M,j\leq N}0.5\times \delta \left(r_{i}-r_{j}=0\right)}{M\times N}$$
  where *M* and *N* denote the number of positive instances and negative instances, respectively; $r_{i}$ and $r_{j}$ indicate the prediction value of positive instances and negative instances, respectively. δ denotes the indication function; when the condition is satisfied, δ = 1, and δ = 0 otherwise;

2. **Log loss**: Log loss is defined in Equation (22), which measures the distance between real labels and prediction scores. A lower log loss indicates better CTR prediction performance. It should be noted that slightly improvement in AUC or decrease in log loss, e.g., at 0.001 level, is be regarded as huge improvement in CTR prediction;
3. ***RI***: Relative improvement (*RI*) measures the improvement of our proposed model over other models. *RI* can be formulated as:
  (24) $$RI_{X}=\frac{\left|X\left(M\right)-X\left(B\right)\right|}{X\left(B\right)}\times 100\%$$
  where *X* denotes the *AUC* or log loss in this paper, *M* represents the proposed method and *B* represents compared models.

#### 4.1.3. Baselines

1. **LR** [21]: LR models first order feature interactions and weight individual features for CTR prediction;
2. **FM** [11]: FM learns the hidden presentation for every feature, then models the second order by the inner product;
3. **AFM** [13]: Based on FM, AFM employs the attention network to model second-order feature interaction importance;
4. **NFM** [33]: NFM is a neural networked version of FM. NFM utilizes a bi-interaction pooling layer for modeling second-order feature interaction; then, MLPs are stacked on the layer to learn high-order feature interactions;
5. **PNN** [32]: PNN models product feature interactions in a product neural network, which is capable of modeling complex feature interactions;
6. **Wide & Deep** [20]: Wide & Deep combines LR and DNNs for modeling low-order and high-order feature interactions, respectively;
7. **DeepFM** [22]: DeepFM replaces the wide component of Wide & Deep with FM to learn more informative feature interactions;
8. **AFN** [28]: AFN implements the feature interaction adaptive order via a logarithmic neural network;
9. **AFN+** [28]: AFN+ is an ensemble model that combines AFN and DNNs to learn feature interactions.

#### 4.1.4. Implementation Detail

We implement the proposed model using Pytorch. The optimization method is set to Adam [37], which is widely used for CTR prediction. Learning rate is 0.001 and 0.0001 for Criteo dataset and Criteo_600K dataset, respectively. The embedding dimension is 10 for all the models, and the batch size is 1024 for all the datasets. The dropout rate is 0.5 for all the models, including DNNs. The layer number of hidden layers is three for all the datasets. Further, the number of hidden units is 64 and 400 for the Criteo_600K and Criteo dataset, respectively. For ALIN, the number of logarithmic neuros is 2000 and 1500 for Criteo600K and Criteo, respectively. The SENet reduction ratio in the proposed ALIN is three and five for Criteo600K and Criteo, respectively.

### 4.2. Comparison Experiment

#### 4.2.1. Effectiveness Comparison

In this section, we compare nine baselines with our proposed models. These comparison models can be divided into four categories: first-order, second-order, high-order, and ensemble model. The first-order feature interaction model (LR) is a linear model that only uses first-order information for feature interaction. Second-order feature interaction models capture the interactions between pairs of features. Higher-order feature interaction models model higher-order feature interactions by various means. Ensemble models combined with DNNs or other modules capture more complex feature interactions. A comparison of model performance is shown in Table 2, from which the following conclusions can be obtained:

1. The performance of LR is the worst among all the comparison models, indicating that first-order interaction is inadequate for CTR prediction;
2. The higher-order model outperforms the second-order model, which shows that finer-grained feature interactions can improve model performance;
3. ALN achieves the best performance among all the high-order models, indicating that it is necessary to consider the importance of feature interactions and the order of feature interactions;
4. ALIN performs best among all the ensemble models, indicating that combining Newton’s identity can reduce noisy information caused by logarithmic neurons.

#### 4.2.2. Efficiency Comparison

In this section, the proposed models were compared with several models on the Criteo dataset in terms of efficiency. The efficiency comparison result is shown in Table 3. The average running time of 20 epochs and the number of parameters were compared based on several models in Table 3. Although AFN has the best performance of efficiency, AFN achieves relatively poor results of *AUC* and log loss compared to other models. It is observed from Table 2 that the value of the AUC for AFN is 0.8087, which is the worst among the four comparison models i.e., AFN, ALN, AFN+, and ALIN. Additionally, the log loss performance of AFN is also the worst among the four models. The performance of ALN improves by 0.001 compared to AFN, with some runtime increase. However, there is not much growth of the number of parameters. From Table 3, we can see that AFN+ has the longest running time and the largest number of parameters. ALIN reduces the running time by 30 s compared to AFN+. In Table 3, ALIN is greatly reduced compared to AFN+ in terms of the number of parameters. From Table 2, we can conclude that ALIN still has a slight improvement in *AUC* and log loss compared to AFN+. In summary, compared with the best baselines models AFN+, ALIN has fewer parameters, faster speed, and better performance.

### 4.3. Hyper-Parameter Experiments

Firstly, we conducted many hyper-parameter experiments on ALN to observe the effects of hyper-parameters in ALN. Subsequently, the optimal parameter setting of ALIN was found based on the ALN parameter settings.

The hyper-parameters of activation in SENet, reduction ratio in SENet, and number of logarithmic neuros were determined by ALN. Additionally, the order of Newton’s identity was determined by ALIN.

#### 4.3.1. The Number of Logarithmic Neuros in ALN

**Figure 5:**
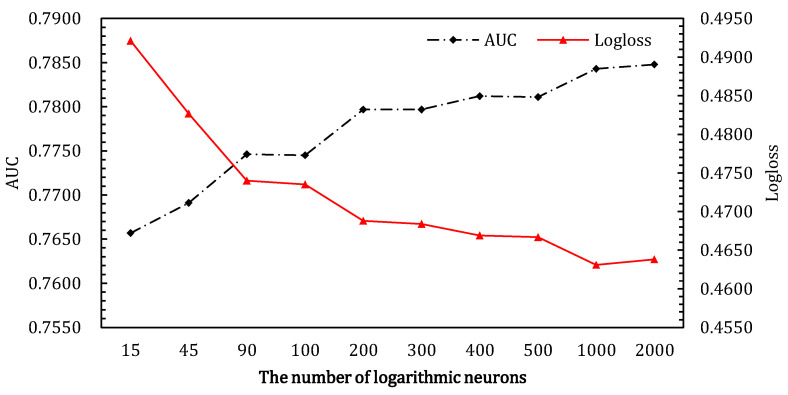
Effects of the number of logarithmic neurons in ALN on Criteo_600K data.

For this section, we only performed hyper-parameter experiments on the Criteo_600K dataset. For the Criteo dataset, we employed the recommended parameter settings in AFN, where the best parameter was 1500. As shown in Figure 5, as the number of logarithmic neurons gradually increases, the performance of the ALN gradually improves, and the effect is optimal when the number of neurons reaches 2000. We observe a significant improvement when the number of logarithmic neurons is 2000 compared with a few dozen neurons. This suggests that more logarithmic neurons can, to some extent, better fit the patterns in the data.

#### 4.3.2. The Type of Activation Functions in SENet

**Figure 6:**
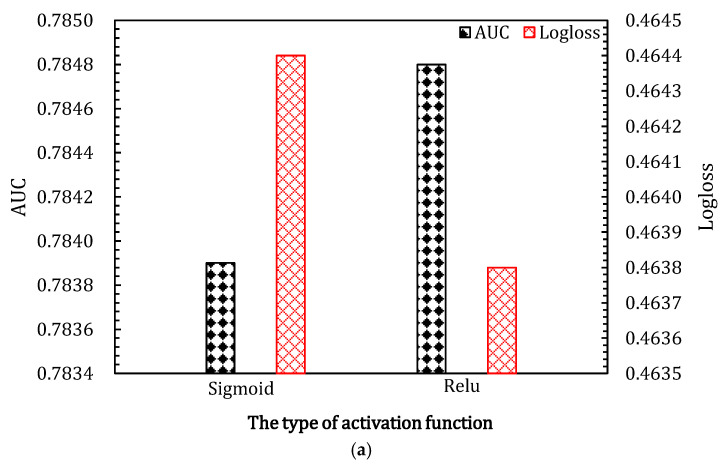

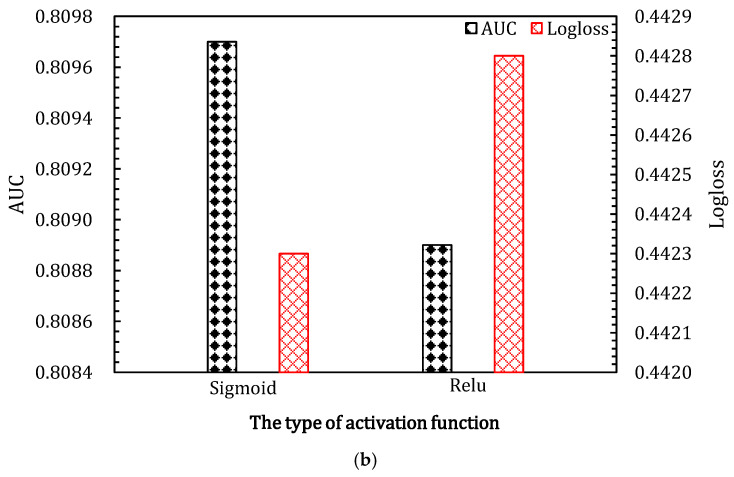
Effects of activation functions in SENet component of ALN on the two datasets. (**a**,**b**) show the results for Criteo_600K dataset and Criteo dataset, respectively. (**a**) Effects of activation functions in SENet component of ALN on Criteo_600K dataset; (**b**) effects of activation functions in SENet component of ALN on Criteo dataset.

The activation function is the key part of the neural network. As shown in Figure 6, different activation functions have different effects on the two datasets. As shown in Figure 6a, the ReLU activation function performs better with the Criteo_600K dataset. However, as shown in Figure 6b, the sigmoid activation function performs better with the Criteo dataset. This indicates that different datasets have different characteristics and need to be fitted with different nonlinear activation functions.

#### 4.3.3. The Reduction Ratio in SENet

**Figure 7:**
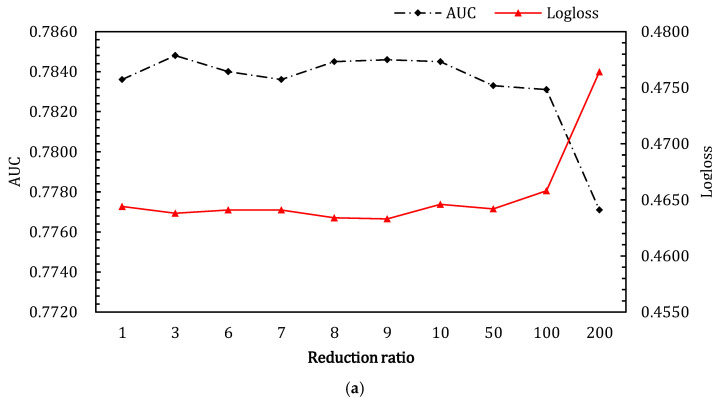

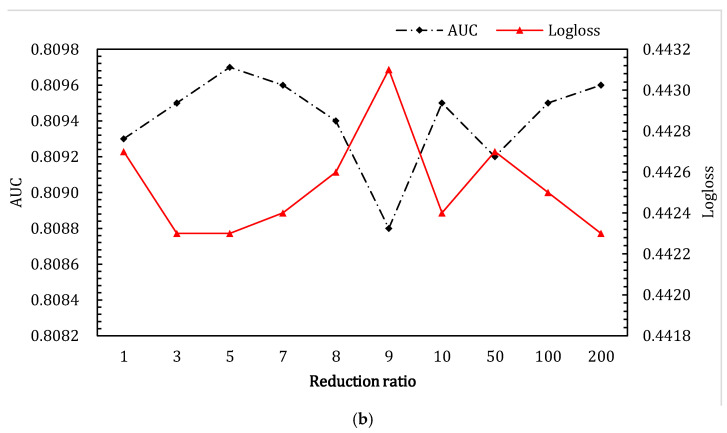
Effects of reduction ratio in SENet of ALN on the two datasets. (**a**,**b**) show the results for Criteo_600K dataset and Criteo dataset, respectively. (**a**) Effects of reduction ratio in SENet component of ALN on Criteo_600K dataset; (**b**) effects of reduction ratio in SENet component of ALN on Criteo dataset.

As shown in Figure 7, the two subfigures demonstrate the effects of different reduction ratio on CTR performance. The results of the reduction ratio hyper-parameter experiments on Criteo_600K are shown in Figure 7a, from which we can observe that performance is optimal when the reduction ratio is three, and the effect gradually decreases with the following ratios. This shows that too much information compression can cause a loss of information, to some extent. In contrast to the former, CTR performance for the Criteo dataset gradually increases until the reduction ratio reaches five, and then it deteriorates before the reduction ratio reaches nine. In conclusion, based on the above results, the reduction ratio is better when it is a small value.

#### 4.3.4. The Order in Newton’s Identity

**Figure 8:**
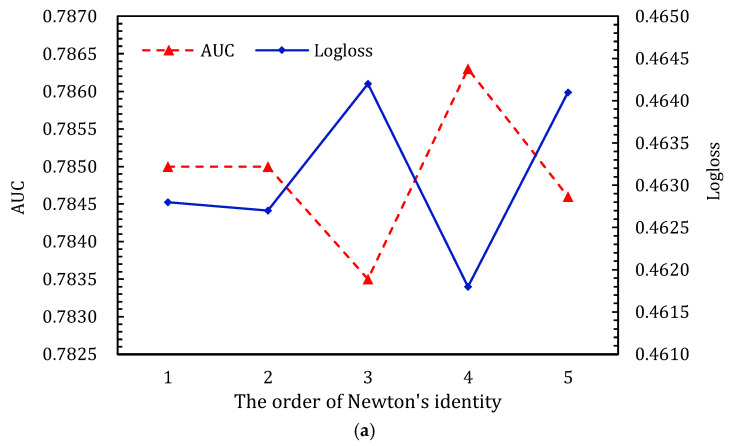

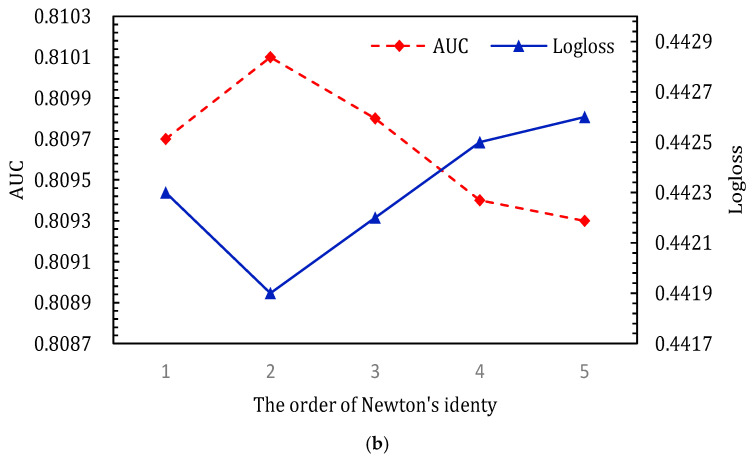
Performance comparison of order of ALIN on the two datasets. (**a**,**b**) show the results for Criteo_600K dataset and Criteo dataset, respectively. (**a**) Effects of order in Newton’s identity component of ALIN on Criteo_600K dataset; (**b**) effects of order in Newton’s identity component of ALIN on Criteo dataset.

The results shown in Figure 8 indicate that different feature interactions need to be supplemented for different datasets. For the Criteo_600K dataset, fourth-order feature interactions are needed as a supplement, while the Criteo dataset needs second-order interactions. In Figure 8a, we can see that ALIN achieves the best performance with the Criteo_600K dataset when the order of feature interactions is four. The performance of ALIN with the Criteo dataset tends to improve until the order is two and then decreases, as shown in Figure 8b. The results for the two datasets demonstrate that a small dataset requires high-order and complex feature interactions to improve performance, while large datasets requires only low-order interactions to improve performance. This is because large datasets have more sufficient information.

### 4.4. Ablation Study

To verify the effectiveness of the individual modules, we designed several ablation experiments, including ALN w/o SE and ALIN w/o NI, which removed the attention mechanism (SENet) based on ALN and Newton’s identity based on ALIN, respectively. The performance of several variants is shown in Table 4, from which the following can be observed:

1. The variant ALIN w/o NI, also known as ALN, outperforms ALN w/o SE, indicating that SENet is beneficial for improving CTR performance. This also shows that it is necessary to consider the importance of feature interactions using SENet;
2. Comparing ALIN w/o NI to ALIN, we can see that ALIN outperforms ALIN w/o NI, which indicates that Newton’s identity can further complement feature interactions to reduce the noise caused by LNN;
3. We can see from the comparison between ALN w/o se and ALIN that the two strategies proposed in this paper significantly enhance CTR performance in both datasets.

## 5. Conclusions

In this paper, we first pointed out the shortcomings of the previous CTR model, which cannot model adaptive-order feature interactions and does not consider the importance of higher-order feature interactions. In addition to this, previous works introduced noise to embedded features during feature interaction. To overcome these drawbacks, the ALIN model was proposed in this paper. The ALIN model uses a logarithmic neural network to model adaptive-order feature interactions, and then uses a squeeze–excitation mechanism to model the importance of higher-order feature interactions. Newton’s identity is combined to complement the feature interactions and compensate for the noise caused by LNN in the embedding. Extensive experiments were conducted on two datasets to show the better performance of this proposed model compared with previous models. Further, hyper-parameter experiments were conducted to observe the effects of hyper-parameters. Several ablation studies were performed to demonstrate the effectiveness of individual components.

## Figures and Tables

**Table 1:** Statistics of the datasets.

Dataset	#Instances	#Train	#Valid	#Test	#Fields
**Criteo_600K**	600,000	480,000	60,000	60,000	39
**Criteo**	45,840,617	36,672,495	4,584,061	4,584,061	39

**Table 2:** Performance comparison between baselines and our proposed model.

Model Type	Model	Criteo_600K				Criteo			
		*AUC*	*RI_AUC_*	Log loss	*RI*_Logloss_	*AUC*	*RI_AUC_*	Log loss	*RI*_Log loss_
**First-order**	**LR**	0.7657	0.0%	0.4858	0.0%	0.7920	0.0%	0.4578	0.0%
**Second-order**	**FM**	0.7804	1.90%	0.4684	3.58%	0.7997	0.97%	0.4517	1.33%
	**AFM**	0.7665	0.10%	0.4809	1.01%	0.7968	0.61%	0.4547	0.68%
**High-order**	**NFM**	0.7838	2.36%	0.4664	3.99%	0.8027	1.35%	0.4488	1.97%
	**PNN**	0.7829	2.25%	0.4724	2.76%	0.8085	2.08%	0.4435	3.12%
	**AFN**	0.7827	2.22%	0.4665	3.97%	0.8087	2.11%	0.4431	3.21%
	**ALN**	**0.7848**	**2.49%**	**0.4638**	**4.53%**	**0.8097**	**2.23%**	**0.4423**	**3.39%**
**Ensemble model**	**Wide & Deep**	0.7827	2.22%	0.4661	4.06%	0.8063	1.81%	0.4460	2.58%
	**DeepFM**	0.7832	2.29%	0.4659	4.10%	0.8049	1.63%	0.4470	2.36%
	**AFN+**	0.7847	2.48%	0.4656	4.16%	0.8100	2.27%	0.4420	3.45%
	**ALIN**	**0.7863**	**2.69%**	**0.4618**	**4.94%**	**0.8101**	**2.29%**	**0.4419**	**3.47%**

**Table 3:** Efficiency comparison on Criteo dataset.

Model	Time (s)	Params
**AFN**	274.95	15,490.25 K
**ALN**	330.20	16,390.25 K
**AFN+**	368.20	25,077.34 K
**ALIN**	339.70	16,390.28 K

**Table 4:** ALIN variant performance.

Variant	Criteo_600K		Criteo	
	*AUC*	Log Loss	*AUC*	Log Loss
ALN w/o SE	0.7827	0.4665	0.8087	0.4431
ALIN w/o NI	0.7848	0.4638	0.8097	0.4423
ALIN	**0.7863**	**0.4618**	**0.8101**	**0.4419**

## Data Availability

Criteo data is available from https://www.kaggle.com/datasets/mrkmakr/criteo-dataset. Criteo_600K dataset is a randomly split dataset of 600,000 samples from the Criteo dataset.
